# Executive functioning in adolescents and adults with Silver-Russell syndrome

**DOI:** 10.1371/journal.pone.0279745

**Published:** 2023-01-20

**Authors:** Mélissa Burgevin, Agnès Lacroix, Fanny Ollivier, Karine Bourdet, Régis Coutant, Bruno Donadille, Laurence Faivre, Sylvie Manouvrier-Hanu, Florence Petit, Christel Thauvin-Robinet, Annick Toutain, Irène Netchine, Sylvie Odent

**Affiliations:** 1 Univ Rennes, LP3C (Laboratoire de Psychologie, Cognition, Comportement et Communication)–EA 1285, FHU GenOMedS, Rennes, France; 2 Univ Angers, Nantes Université, LPPL, SFR CONFLUENCES, Angers, France; 3 Service de Pédiatrie, CHRU Brest, Brest, France; 4 Service Endocrinologie Pédiatrique, CHU Angers, Angers, France; 5 Endocrinologie, Service du Pr Christin-Maitre, Hôpital Saint Antoine, Sorbonne Université, Assistance Publique-Hôpitaux de Paris, Paris, France; 6 INSERM–University of Bourgogne Franche-Comté, UMR 1231 GAD Team, Genetics of Developmental Disorders, FHU TRANSLAD, CHU Dijon Bourgogne, Dijon, France; 7 CHU Dijon, Centre de Référence Maladies Rares Anomalies du Développement et Syndromes Malformatifs, Centre de Génétique, FHU TRANSLAD, CHU Dijon Bourgogne, Dijon, France; 8 CHU Lille, Centre de Référence Maladies Rares Labellisé Pour les Anomalies du Développement Nord-Ouest, Clinique de Génétique, Lille, France; 9 Univ Lille, EA7364 –RADEME–Maladies Rares du Développement Embryonnaire et du Métabolisme : du Phénotype au Génotype et à la Fonction, Lille, France; 10 CHU Lille, Clinique de Génétique Guy Fontaine, Lille, France; 11 Service de Génétique, Centre Hospitalier Universitaire, UMR 1253, iBrain, Université de Tours, Inserm, FHU GenOMedS, Tours, France; 12 Sorbonne Université, INSERM UMR_S 938, Centre de Recherche Saint Antoine, APHP, Hôpital Armand Trousseau, Explorations Fonctionnelles Endocriniennes, Paris, France; 13 Service de Génétique Clinique, Centre Référence Anomalies du Développement CLAD Ouest, Univ Rennes, CNRS, INSERM, IGDR (Institut de Génétique et Développement de Rennes), UMR 6290 ERL 1305, FHU GenOMedS, Rennes, France; Radboud University: Radboud Universiteit, NETHERLANDS

## Abstract

Silver-Russell syndrome (SRS) is a rare imprinting disorder characterized by prenatal and postnatal growth retardation. The two principal causes of SRS are loss of methylation on chromosome 11p15 (11p15 LOM) and maternal uniparental disomy of chromosome 7 (UPD(7)mat). Knowledge of the neuropsychological profile of SRS remains sparse and incomplete even if several difficulties related to attention and learning have been reported both in the literature and by patients with SRS. These difficulties could be the result of troubles in different cognitive domains, but also of executive dysfunction. Nevertheless, executive functioning has never been investigated, even though executive functions play an essential role in psychological development, and are extensively involved in daily life. The present study explored the executive functioning of individuals with SRS due to UPD(7)mat or 11p15 LOM. A battery of executive tasks assessing cognitive flexibility, inhibitory control, and working memory, together with a task assessing sustained attention, was administered to 19 individuals with SRS (13–39 years) and 19 healthy controls. The Behavior Rating Inventory of Executive Function was also completed by the participants’ families. The results showed that participants with SRS had similar performance (z-scores) to our controls, in a context of normal intellectual efficiency. Group comparisons with Bayesian statistics showed a single difference between the 11p15 LOM and control groups: the completion time for part A of the Trail Making Test appeared to be longer in the 11p15 LOM group than in the control group. However, at the clinical level, several participants with SRS had clinically significant scores on various measures of EFs. Thus, the cognitive phenotype of SRS did not appear to be characterized by executive dysfunction, but individuals with SRS could be at high risk of developing executive dysfunction or attention-deficit/hyperactivity disorder. These results provide new insights into the neuropsychological profile of individuals with SRS.

## Introduction

*Executive functions* (EFs)–also referred to as *cognitive control* or *executive control* in the literature–are traditionally defined as a set of high-level cognitive processes that engage, direct, or coordinate other cognitive processes [1]. EFs typically allow individuals to intentionally control and regulate their thoughts and actions to achieve a particular goal [2, 3]. They are important when adapting to the demands of the environment or new situations, and are elicited when routines, automatisms or overlearned cognitive skills no longer suffice for carrying out an action or activity [2, 4]. Although there is currently no real consensus about the definition, components, and model of EFs, there are nevertheless points of convergence [5]. Most studies acknowledge that EFs include, but are not limited to, three interrelated core skills: inhibitory control, cognitive flexibility or set shifting, and working memory or updating [2, 4, 6, 7]. The theoretical distinctions between working memory versus updating and set shifting versus cognitive flexibility are still debated in the literature [8, 9]. *Inhibitory control* refers to the ability to deliberately curb, suppress, or more generally control goal-irrelevant stimuli, and cognitive or behavioral responses [2]. *Cognitive flexibility* is the ability to appropriately adapt one’s behavior to a changing environment. This includes set shifting, namely the ability to switch between multiple tasks or mental sets [9]. *Working memory* refers to the ability to process, manipulate and update information during cognitive activities [10]. Several other cognitive processes are more or less consensually associated with EFs, including attention, planning/organization, initiation, self-regulation, reasoning and problem-solving [2, 11]. Some authors distinguish between *hot* and *cool* executive processes, depending on the situations in which they are engaged [12]. *Hot* processes support the use of EFs in motivational and affectively or emotionally significant tasks. By contrast, inhibitory control, cognitive flexibility, working memory, and planning are regarded as *cool* processes, used in affectively neutral contexts, in relatively abstract or decontextualized tasks [1]. In parallel with the maturing of prefrontal networks, EFs emerge in the first few months of life but only reach full maturity in adulthood [6]. Their development is therefore both early, dynamic and prolonged. EFs are relatively undifferentiated up to the age of 5 years [13]. After 6 years, they gradually separate into specialized and partially independent executive processes. According to Diamond’s hierarchical and integrative model, inhibitory control and working memory are the first processes to be differentiated [2]. These two components then allow for the gradual strengthening of cognitive flexibility. Finally, the development of these three basic skills allows higher-level components such as planning, reasoning and problem-solving to be differentiated. Several sociodemographic factors are thought to play a role in the development of EFs, including sex, educational attainment, parents’ education level, and culture [14, 15]. To date, there is no established consensus on the precise effects of these different factors. However, education level is known to influence cognitive and executive performance [16–18] and is generally positively correlated with performance on executive tasks [16–19].

The developmental particularities of EFs, as well as the vulnerability of frontosubcortical networks, constitute a risk factor for the occurrence of executive dysfunction. As a result, disturbances of EFs are present in many pathologies such as acquired lesions and acquired or genetic neurodevelopmental disorders [20–27]. These executive disorders can have significant repercussions on the lives of patients and their families [28]. EFs are also known to play an important role in human development [29], particularly psychological development, achievement, and social integration [2]. It is therefore essential to assess EFs, particularly to understand neuropsychological phenotypes, not least in genetic syndromes [30].

Silver-Russell syndrome (SRS; OMIM #180860) is a rare imprinting disorder characterized by prenatal and postnatal growth retardation. Its prevalence at birth is estimated to be 1/15 866, with boys and girls equally affected [31, 32]. The two principal causes are maternal uniparental disomy of chromosome 7 (UPD(7)mat), which is identified in about 5–10% of cases, and, in 50% of individuals, loss of methylation on chromosome 11p15 (11p15 LOM), which contains imprinted fetal growth factors [33, 34]. However, it currently remains unknown for many patients, so the diagnosis of SRS is primarily based on clinical signs. The clinical diagnosis is currently based on the Netchine-Harbison clinical scoring system (NH-CSS), where patients have to meet at least four of the following six clinical criteria: 1) born small for gestational age, 2) postnatal growth retardation, 3) relative macrocephaly at birth, 4) prominent forehead at the ages 1–3 years; 5) body asymmetry and 6) feeding difficulties and/or low body mass during early childhood [35, 36]. A recently published study demonstrated that some of these criteria are no longer discriminatory diagnostic features in adulthood [37]. Molecular confirmation is therefore necessary for these patients. In addition to limited growth, individuals with SRS may have other clinical features, such as early puberty, genital anomalies and metabolic disorders [36]. Children may benefit from growth hormone (GH) treatment to improve their physical condition, and gonadotropin-releasing hormone analogue (GnRHa) treatment to slow down puberty in some cases. Genotype-phenotype studies show that patients with 11p15 LOM have a more severe phenotype than those with UPD(7)mat, but the latter exhibit more frequent neurocognitive and behavioral disorders [38, 39].

The neuropsychological profile of individuals with SRS is generally characterized by intellectual efficiency in the medium to the low normal range [40]. IQ seems to be significantly impaired when individuals with clinical SRS are compared with a control group or with typical siblings [41, 42]. The incidence of intellectual impairment in the clinical SRS also appears to be higher than in the general population [43, 44]. Genotypically, the IQ of patients with UPD(7)mat is significantly lower than the one of patients with 11p15 LOM [42, 45]. Developmental delays and cognitive difficulties have also been reported in several SRS cases, ranging from mild motor or language delays to more persistent and severe difficulties [39]. Children with SRS, particularly those with UPD(7)mat, may have learning difficulties, particularly in reading, writing, and mathematics [43, 46]. Children with UPD(7)mat are also at increased risk of developing autism spectrum disorders, verbal dyspraxia, and myoclonus-dystonia syndrome [35, 45, 47, 48]. However, although difficulties have been reported among patients with SRS, they have rarely been thoroughly investigated. For example, little interest has been given to EFs and attention in SRS so far, even though impulsivity and reduced attention/concentration, attention deficit disorder (ADD) or attention-deficit/hyperactivity disorder (ADHD) have been observed in some children with SRS [39, 46, 49–51]. ADHD is characterized by executive and attentional impairments. A recent study relates that the majority of adults with 11p15 LOM reported daily difficulties, especially with attention/concentration and learning [40]. Potential co-morbidity with learning disabilities and/or ADHD was also suggested in anotherone [52]. Very recently, researchers found that total brain volume was unchanged in patients with SRS compared with a control group [42]. However, gray-matter volume in the frontal and temporal lobes and globus pallidus was reduced in patients with SRS, both those with UPD(7)mat and those with 11p15 LOM. These structural brain characteristics could be associated with specific cognitive peculiarities, especially in executive and attentional functions, for patients with SRS, even in the absence of impaired overall intellectual efficiency.

The international consensus on the diagnosis and management of SRS recommends neuropsychological assessments at different stages of development to identify potential problems and provide appropriate interventions [36]. Although several studies provide knowledge about the difficulties faced in SRS, they need to be supported and confirmed in individuals diagnosed according to the clinical criteria of the NH-CSS and confirmed by molecular diagnosis. Therefore, in light of the difficulties reported in SRS, and more specifically attentional difficulties, as well as the vulnerability of executive functions observed in different genetic syndromes, we chose to conduct an exploratory study to investigate executive and attentional functions in SRS. Our objective was to determine which abilities are preserved and which ones are impaired in SRS patients. The tested hypothesis was that, compared with control groups, the SRS group would perform more poorly on executive tasks (e.g., attention, inhibitory control) and report more difficulties in daily life.

## Materials and methods

### Design

We conducted this cross-sectional study as part of a larger research project on the life course and neuropsychological profile of adolescents and adults with SRS [40, 53]. This study involving human participants was reviewed and approved by the ethics committee of Rennes University Hospital, France (no. 15.123, 29 December 2015). It was also conducted in accordance with the Declaration of Helsinki. All participants (and their parents, in the case of adolescents) provided their written informed consent to participate in this study.

### Participants

Nineteen individuals aged 13–39 years, who have been diagnosed with SRS in accordance with the NH-CSS, confirmed by molecular analysis, participated in this study [35]. This group included three participants with UPD(7)mat and 16 with 11p15 LOM. They were recruited between 2016 and 2018 in France via a call for participation in the study. This call was relayed to patients with SRS and their families by 1) the referral center at Trousseau Children’s Hospital in Paris, 2) geneticists and endocrinologists from various French hospitals and reference centers for developmental anomalies affiliated with the AnDDI-Rares disease healthcare network, FIRENDO rare endocrine diseases network and 3) two French patient organizations. Only patients with a clinical diagnosis of SRS confirmed by molecular analysis were included in this study. Indeed, although the NH-CSS assists in diagnosis in line with standards recommended by the international consensus [36], the accuracy of the clinical diagnosis may depend on the experience of the medical investigator [54].

Nineteen healthy volunteers were recruited to form a control group. The control group was matched for age, sex, and education level to the SRS group. Inclusion criteria for these participants were not having SRS, no history of psychiatric or neurodevelopmental disorders, no learning disabilities, or other neurological disorders as well as no GH or GnRHa treatment in their lifetime. Demographic and clinical characteristics of the SRS and control groups are summarized in Table 1. The total SRS group and control group did not differ in sociodemographic characteristics: sex (Fisher’s exact test, *p =* 1.000), age (*U* = 169, *p =* .745), education level (*t*(36) = 0.06, *p* = .956), parents’ education level (*t*(34) = 0.79, *p* = .435). The 11p15 LOM group did not differ either from the control group: sex (Fisher’s exact test, *p =* 1.000), age (*U* = 116.5, *p =* .241), education (*t*(33) = 0.65, *p* = .522), parents’ education level (*t*(31) = 0.90, *p* = .376).

**Table 1:** Sociodemographic characteristics of SRS and control groups.

	SRS group			Control group
	Total group	11p15 LOM group	UPD(7)mat group	
Number of participants	19	16	3	19
Sex				
Males	10	9	1	10
Females	9	7	3	9
Age (years)				
Mean (*SD*)	20.05 (7.21)	21.31 (7.19)	13.33 (0.58)	18.79 (5.14)
Range	13–39	13–39	13–14	13–31
Educational level (years)				
Mean (*SD*)	11.26 (2.98)	11.94 (2.74)	7.67 (0.58)	11.32 (2.91)
Range	7–17	8–17	7–8	7–17
Parental educational level (years)				
Mean (*SD*)	12.45 (3.10)	12.28 (3.25)	13.33 (2.47)	13.24 (2.86)^a^
Range	5–17	5–17	10.50–15	8.5–17^a^

*Note*. SRS = Silver-Russell syndrome; 11p15 LOM = loss of methylation on chromosome 11p15; UPD(7)mat = maternal uniparental disomy of chromosome 7; *SD* = standard deviation.

^a^ For parental educational level (years), *N* = 16 for the control group.

### Measures

#### Participants’characteristics and demographic data

The collected sociodemographic features included age, sex, education level and parents’ education level (see Table 1). Intellectual functioning was assessed by administering the French-language version of the Wechsler Intelligence Scale for Children, fourth (WISC-IV) or fifth edition (WISC-V), to adolescents aged 13–16 years, and the Wechsler Adult Intelligence Scale, fourth edition (WAIS-IV) to individuals aged 17 years or above [55–57]. We calculated the Full-Scale Intellectual Quotient (FSIQ; *M* = 100, SD = 15), the Verbal Comprehension Index (VCI), the performance/nonverbal IQ (PIQ; the Perceptual Reasoning Index in WAIS-IV and WISC-IV, and the Fluid Reasoning Index in WISC-V), the Working Memory Index (WMI), and the Processing Speed Index (PSI).

The total SRS group and control group did not differ on FSIQ (*t*(36) = 1.34, *p* = .190), VCI (*t*(30.80) = 0.37, *p* = .717), PIQ (*t*(36) = 1.23, *p* = .228), WMI (*U* = 213, *p =* .349), PSI (*t*(36) = 1.07, *p* = .290) (see Table 2). The 11p15 LOM group did not differ from the control group (Full-Scale IQ, *t*(23.53) = 1.19, *p* = .245; VCI, *t*(33) = 0.93, *p* = .927; PIQ, *t*(33) = 1.22, *p* = .232; WMI, *t*(33) = 1.76, *p* = .088; PSI, *t*(33) = 1.10, *p* = .280). Intra-individual analyses were performed on part of the data in a previous study [40].

**Table 2:** Full-scale IQ and the four index scores of SRS and control groups.

	SRS group			Control group (*N* = 19)
	Total group	11p15 LOM group	UPD(7)mat group	
	(*N* = 19)	(*N* = 16)	(*N* = 3)	
Full-Scale IQ				
Mean (*SD*)	101.63 (17.36)	101.75 (18.08)	101.00 (16.09)	107.89 (10.75)
Range	71–127	71–127	86–118	92–128
VCI				
Mean (*SD*)	113.16 (17.37)	114.44 (17.66)	106.33 (17.10)	114.89 (11.22)
Range	79–139	79–139	95–126	94–135
PIQ				
Mean (*SD*)	97.21 (15.40)	96.81 (16.59)	99.33 (8.02)	102.74 (12.18)
Range	74–128	74–128	91–108	84–126
WMI				
Mean (*SD*)	96.58 (16.88)	94.63 (17.47)	107.00 (9.17)	103.95 (13.87)
Range	63–117	63–117	97–115	80–128
PSI				
Mean (*SD*)	95.21 (12.83)	94.81 (13.51)	97.33 (10.26)	99.47 (11.62)
Range	69–114	69–114	86–106	72–119

*Note*. SRS = Silver-Russell syndrome; 11p15 LOM = loss of methylation on chromosome 11p15; UPD(7)mat = maternal uniparental disomy of chromosome 7; *SD* = standard deviation, IQ = Intelligence Quotient; VCI = Verbal Comprehension Index; PIQ = Performance IQ; WMI = Working Memory Index; PSI = Processing Speed Index.

The Reliable Digit Span (RDS) was calculated for each group to ensure the performance validity on this neuropsychological assessment. The RDS was calculated by summing the longest forward and backward raw digit spans in which both trials were correctly produced on the Digit Span subtests of the Wechsler scales [58, 59]. For the SRS group, the average RDS performance was 8.11 (*SD* = 1.56, Range 5–11). Based on the previously established adult cut-off ≤ 7, 84.21% of the sample passed; at a cut-off ≤ 6, 89.97% of the sample passed which approaches a 90% pass rate recommended in the literature [58]. For the 11p15 LOM group (*M* = 8.19, *SD* = 1.49, Range 5–11), at the cut-off ≤ 7 and ≤ 6, 87.5% and 93.75% of the sample passed. For the UPD(7)mat group, the RDS average was 7.67 (*SD* = 2.31, Range 5–9). At the cut-off ≤ 7 and ≤ 6, 66.67% of the sample passed. For the control group (*M* = 9.63, *SD* = 2.03, Range 7–15), 100% of the sample passed at the cut-off ≤ 7 and ≤ 6.

### Executive functions

EFs were tested using six cognitive tests: d2-R test, Digit span subtest, Trail Making Test (TMT), Stroop test, Verbal fluency test, and Modified Card-Sorting Test (MCST) [55–57, 60–65]. The Behavior Rating Inventory of Executive Function (BRIEF) questionnaires for children and adults were also used [66–69].

The *d2-R test*, a speeded paper-and-pencil task, measures selective and sustained attention, concentration, and impulsivity. Participants were each given a sheet of paper with 14 rows of 57 characters. Each character was either the letters *p* or *d*, with 1–4 dashes above and/or below. Participants had to locate and cross out all the target characters (letter *d* with two dashes, either one above and one below, or both either above or below) as quickly as possible and without errors. To achieve this, they had to process each row within 20 seconds, and all the rows were processed in succession without interruption. This task took 4 minutes and 40 seconds in total. The raw scores of five parameters of the d2-R were analyzed: the total number of items processed (i.e., processing speed), the total number of responses minus total number of errors (i.e., sustained attention/concentration), percentage of error (i.e., accuracy), the omission errors, and the commission errors.

The *Digit span subtest* (WISC-IV, WISC-V or WAIS-IV) was used to measure verbal working memory. Series of numbers were presented verbally, and participants had to repeat the series of digits in the reverse order (Digit span backward). The series of numbers gradually increased if participants continued to answer correctly. We analyzed performances on the backward spans in this study.

The *Trail Making Test* (TMT) comes in two parts: A (TMT-A) and B (TMT-B). In Part A, participants were asked to link numbers from 1 to 25 in ascending order. In Part B, they had to link numbers (1–13) and letters (A-L) in alternating ascending and alphabetical order (1A, 2B, etc.). In both parts, participants had to link the numbers or letters and numbers as quickly as possible. Part A measures attention, visual scanning and psychomotor speed, while Part B assesses the ability to flexibly shift course during an activity. In this study, completion times (in seconds) for TMT-A and TMT-B were recorded. Cognitive flexibility was assessed by calculating the difference in completion time between Parts B and A of the TMT (B-A).

The *Stroop test* is an adapted version of the original Stroop test, assessing inhibitory control [63]. All three test conditions were administered. In the first condition (naming), participants had to name the color of 100 rectangles (red, green, blue) as quickly as possible. In the second condition (reading), they had to read as quickly as possible 100 names of color (red, green, blue) printed in black. In the last condition (interference), participants had to name the color of the ink in which 100 names of color were printed (e.g., saying *red* when the word *green* was printed in red ink). This condition requires automatic reading to be inhibited, and participants had to perform it as quickly as possible. Inhibitory processing was assessed by calculating the difference between the time taken to perform the trials in the interference condition and the time taken to perform them in the naming condition.

The *Verbal fluency test*, which measures the ability to generate words quickly, was used to assess spontaneous cognitive flexibility. Participants were asked to produce as many French words as possible in 120 seconds. They were asked to generate words starting with the letter *p* (phonemic fluency), and words belonging to the animal category (semantic fluency). For phonemic fluency, participants were instructed to avoid proper names, same-root names, and repetitions. For semantic fluency, they had two restrictions: same-root names and repetitions. Scores were calculated by subtracting incorrect responses and repeated words from the total number of responses.

The *Modified Card-Sorting Test* is a multicomponent task that specifically measures reactive flexibility, perseverative responding, and categorization. It is a simplified version of the Wisconsin Card Sorting Test, which contains two sets of 24 cards and no ambiguous ones [70]. Participants had to match each of the 48 cards to one of four target cards. Cards could be matched by color, form, or number of symbols. Participants were free to choose the initial sorting category. Then, they had to guess the sorting principle solely based on the examiner’s feedback (“yes” or “no”). In this study, we used an adapted version of the MCST: after six correct responses, the sorting principle was changed without prior warning, unlike the initial version (“Now the rules have changed”). The number of categories completed, total errors and perseverative errors were counted.

The parent form of the French version of the BRIEF was used to assess adolescents’ EFs in everyday life [67]. The BRIEF for children and adolescents aged 5–18 years includes 86 behavioral items. For each item, the parent indicates the frequency of the behavior on a 3-point Likert scale (*Never*, *Sometimes*, *Often*). The BRIEF consists of eight clinical subscales that allow three indices to be calculated: the Behavioral Regulation Index (BRI), encompassing the Inhibit, Shift and Emotional control subscales; the Metacognition Index (MI), which includes the Initiate, Working memory, Plan/Organize, Organization of materials and Monitor subscales; and the Global Executive Composite (GEC), the sum of all the clinical subscales. For adults, we used the informant-report forms of the French version of the BRIEF for adults aged 18–90 years (BRIEF-A) [69]. The BRIEF-A is composed of 75 items that measure various aspects of executive functioning. Nine clinical subscales and three indices to be calculated: the BRI, which includes the Inhibit, Shift, Emotional control, and Self-monitor subscales; the MI, containing the Initiate, Working memory, Plan/Organization, Task monitor, Organization of materials subscales; and the GEC. In this study, we considered the BRI, MI and GEC *T*-scores (*M* = 50, *SD* = 10) and the Inhibit, Shift, Working memory and Plan/Organize subscales *T*-scores for adolescents and adults. *T*-scores above 65 were considered clinically significant.

### Procedure

A neuropsychological assessment was conducted by a psychologist. All participants were tested individually in their own homes. Before the assessment, a semi-structured interview was conducted to collect sociodemographic characteristics and medical data for the SRS group. Participants first completed the d2-R test, followed by the Wechsler Intelligence Scale (including Digit span subtest), TMT, Stroop test, Verbal fluency test, and finally the MCST. The assessment lasted 3 hours on average, with many breaks, depending on the needs of each participant.

### Statistical analyses

Statistical analyses were performed using JASP (version 0.16.4, Intel) [71]. Descriptive statistics were used to characterize samples. The normality of distribution was assessed with the Shapiro-Wilk normality test and confirmed by an inspection of the Q-Q plot. Levene’s test was conducted to assess the equality of variances. For sociodemographic and intellectual data, we used either independent *t*-tests or Welch’s *t*-tests to compare continuous variables with a normal distribution. Mann-Whitney *U*-tests were used for continuous variables with non-normal distributions. Fisher’s exact test was used for categorical variables. For intellectual scores, a Bonferroni correction was applied to reduce the chances of obtaining false-positive results (Type I errors). The Bonferroni correction was *p* < 0.01 for the Full-Scale IQ and the four index scores (VCI, PIQ, WMI, PSI).

For EF scores, z-scores of UPD(7)mat subjects and z-scores of the 11p15 LOM group were calculated according to the mean and standard deviation of the control groups. Group comparisons were performed to determine the differences between SRS and control groups. Due to the small sample size of the UPD(7)mat group (*N* = 3), comparisons were made only between 11p15 LOM and control groups. To this end, we used the Bayesian Mann-Whitney *U*-tests framework proposed by Jeffreys [72]. Bayes factors (BF_10_) were calculated to quantify the relative probability of the hypothesis (H_1_, the model including the effect of interest) and the null (H_0_, the same model without this effect). By convention, a BF of 1–3 is considered negligible evidence for H_1_, 3–10 indicates moderate evidence, 10–30 is strong, 30–100 is very strong, and a BF > 100 is extreme evidence. Alternatively, a BF of 0.33–1 is considered weak to negligible evidence in favor of H_0_, 0.1–0.33 is moderate evidence, and < 0.1 is strong evidence [72, 73]. A Cauchy distribution with a scale *r* = $1/\sqrt{2}$ (0.707) as a default choice for the prior distribution of effect size was used [74]. Dataset and codebook have been deposited in Open Science Framework with the identifier: https://doi.org/10.17605/osf.io/32zjt (https://osf.io/32zjt/).

## Results

Table 3 provides an overview of the performance of the 11p15 LOM group compared with the control group, showing the *z*-scores for the 11p15 LOM group’s performance on each task, calculated according to the mean and standard deviation of the control group.

**Table 3:** Z-scores of participants with 11p15 LOM group calculated for each executive function task based on the mean and standard deviation of the control group.

	Mean (SD)	Range	% clinically significant^a^
d2-R (n = 15)			
Processing speed	-0.35 (1.23)	-2.35 to 2.38	13
Sustained attention/concentration	-0.52 (1.44)	-2.44 to 2.59	27
Accuracy	0.77 (2.18)	-1.07 to 6.83	27
Omission errors	0.34 (1.71)	-1.04 to 5.42	13
Commission errors	1.55 (2.20)	-0.32 to 5.68	53
**Digit span subtest (n = 16)**			
Span backward	-0.33 (1.43)	-2.41 to 2.59	6
**Trail Making Test (n = 16)**			
Time Part A	1.44 (1.72)	-1.01 to 4.44	38
Time Part B	1.18 (2.38)	-1.35 to 7.57	25
Time Part B-A	0.70 (2.38)	-1.47 to 7.04	25
**Stroop (n = 16)**			
Interference time	0.11 (1.51)	-1.83 to 4.23	13
**Verbal fluency (n = 16)**			
Letter *p*	-0.43 (1.26)	-3.99 to 1.95	6
Animal category	0.02 (1.35)	-1.76 to 2.17	6
**Modified Sorting Card Test (n = 16)**			
Categories completed	0.02 (0.80)	-1.32 to 0.46	0
Total errors	-0.06 (0.94)	-1.00 to 2.06	6
Perseverative errors	-0.10 (1.77)	-0.77 to 5.89	13
**BRIEF/BRIEF-A questionnaire (n = 16)**			
Global Executive Composite	-0.01 (0.87)	-1.41 to 1.53	0
Behavioral Regulation Index	-0.30 (0.69)	-1.41 to 1.16	0
Metacognition Index	0.19 (1.10)	-1.05 to 1.94	6
Inhibit subscale	-0.19 (0.46)	-1.04 to 0.82	0
Shift subscale	-0.19 (0.78)	-1.15 to 1.30	0
Working memory subscale	0.43 (1.30)	-1.06 to 3.00	31
Plan/Organize subscale	0.13 (0.96)	-0.91 to 1.49	0

Note.

^a^ z-score ≥ 1.65; SD = standard deviation; For accuracy, omission errors, and commission errors of the d2-R, the times of TMT-A, TMT-B, and Part B-A, interference time of the Stroop, total errors and perseverative errors of the MCST, and the indices and subscales of BRIEF/BRIEF-A questionnaire, high z-scores reflect poor performances of the 11p15 LOM group.

Overall, results show that the 11p15 LOM group performances are lower than the one of the control group, even if high interindividual variability exists. In particular, several individuals (n = 6) had clinically significant scores on the d2-R, the TMT, and the working memory subscale of the BRIEF/BRIEF-A questionnaire. The majority of individuals with clinical scores were adults with 11p15 LOM. Clinically significant scores were also observed for participants with UPD(7)mat (see Table 4).

**Table 4:** Z-scores of participants with UPD(7)mat group calculated for each executive function task based on the mean and standard deviation of the control group.

	Subject 1	Subject 2	Subject 3
d2-R			
Processing speed	-2.64*	-0.91	-0.17
Sustained attention/concentration	-2.61*	-0.96	-0.05
Accuracy	-1.18	0.10	-0.27
Omission errors	-1.19	-0.60	-0.60
Commission errors	-0.32	5.68*	3.68*
**Digit span subtest**			
Span backward	-0.74	0.93	-1.58
**Trail Making Test**			
Time Part A	0.16	1.19	-0.36
Time Part B	3.30*	-0.04	-1.35
Time Part B-A	3.96*	-0.66	-1.47
**Stroop**			
Interference time	4.37*	0.28	1.34
**Verbal fluency**			
Letter *p*	-3.2*	0.17	-1.42
Animal category	-1.76*	0.44	0.91
**Modified Sorting Card Test**			
Categories completed	-3.11*	-3.11*	0.46
Total errors	2.06*	0.17	-0.53
Perseverative errors	2.56*	0.56	-0.11
**BRIEF questionnaire**			
Global Executive Composite	0.67	1.24	1.05
Behavioral Regulation Index	-0.86	-0.31	1.16
Metacognition Index	1.47	1.85*	0.82
Inhibit subscale	-0.07	0.37	2.40*
Shift subscale	-1.23	0.15	-0.01
Working memory subscale	1.65*	1.08	1.65*
Plan/Organize subscale	1.03	1.86*	0.84

Note.

* indicate clinically significant z-scores (z-score ≥ 1.65); For accuracy, omission errors, and commission errors of the d2-R, the times of TMT-A, TMT-B, and Part B-A, interference time of the Stroop, total errors and perseverative errors of the MCST, and the indices and subscales of the BRIEF questionnaire, high z-scores reflect poor performances of subjects with UPD(7)mat.

### Comparison between 11p15 LOM and control groups

Because of the non-normal distribution of the data, Bayesian Mann-Whitney *U*-tests were used to test the null and the alternative hypothesis. The null hypothesis postulated that there was no difference in EF scores between the 11p15 group and the control group. On account of the nature of the data, we had two one-sided alternative hypotheses: 1) the 11p15 LOM group had smaller scores on executive tasks than the control group (BF_+0_), and 2) the 11p15 LOM group had higher scores on executive tasks than the control group (BF_-0_). Indeed, depending on the nature of the data (e.g., response times of TMT), lower performance corresponded to higher scores. The results are shown in Table 5.

**Table 5:** Comparison of executive function scores between 11p15 LOM and control groups.

	11p15 LOM group	Control group	BF_+0_	BF-_0_	W
	(*N* = 16)	(*N* = 19)			
	Mean (*SD*)	Mean (*SD*)			
d2-R					
Processing speed	145.00 (33.26)^a^	154.53 (27.05)	0.73		174.00
Sustained attention/concentration	132.60 (34.87)^a^	145.26 (24.26)	1.04		182.50
Accuracy	8.76 (8.45)^a^	5.76 (3.87)		1.06	115.00
Omission errors	11.40 (11.61)^a^	9.11 (6.81)		0.59	130.00
Commission errors	0.93 (1.10)^a^	0.16 (0.50)		2.97	80.50
**Digit span subtest**					
Span backward	4.50 (1.71)	4.89 (1.20)	0.85		190.00
**Trail Making Test**					
Time Part A	37.88 (13.25)	26.79 (7.71)		5.02	76.50
Time Part B	85.19 (43.49)	63.68 (18.27)		1.47	109.00
Time Part B-A	47.31 (35.59)	36.89 (14.93)		0.39	149.00
**Stroop**					
Interference time	43.56 (21.45)	42.00 (14.20)		0.28	168.00
**Verbal fluency**					
Letter *p*	21.00 (6.36)	23.16 (5.05)	0.72		176.00
Animal category	32.31 (8.56)	32.21 (6.36)	0.43		166.00
**Modified Sorting Card Test**					
Categories completed	5.75 (0.45)	5.74 (0.56)	0.36		156.00
Total errors	9.00 (3.98)	9.26 (4.25)		0.28	157.00
Perseverative errors	1.31 (2.65)	1.16 (1.50)		0.25	174.00
**BRIEF/BRIEF-A questionnaire**					
Behavioral Regulation Index	47.06 (7.54)	50.35 (10.86)^b^		0.20	153.50
Metacognition Index	50.31 (11.72)	48.24 (10.70)^b^		0.37	129.00
Global Executive Composite	48.75 (9.15)	48.88 (10.55)^b^		0.32	128.00
Inhibit subscale	46.63 (5.18)	48.76 (11.33)^b^		0.31	138.50
Shift subscale	49.75 (9.62)	52.12 (12.25)^b^		0.25	146.50
Working memory subscale	51.19 (11.55)	47.41 (8.86)^b^		0.60	117.00
Plan/Organize subscale	50.31 (10.35)	48.88 (10.82)^b^		0.45	125.50

*Note*. 11p15 LOM = loss of methylation on chromosome 11p15; *SD* = standard deviation; BF_+0_ = Bayes factor that quantifies evidence for the one-sided alternative hypothesis that group *Control* is greater than group *11p15 LOM*, relative to the null hypothesis; BF_-0_ = Bayes factor that quantifies evidence for the one-sided alternative hypothesis that group *Control* is smaller than group *11p15 LOM*, relative to the null hypothesis. Results are based on a data augmentation algorithm with 5 chains of 1000 iterations, with 1 seed for repeatability.

^a^ For all measures of the d2-R test, *N* = 15 for the 11p15LOM group.

^b^ For all index and subscales of the BRIEF/BRIEF-A questionnaire, *N* = 17 for the control group.

Comparing groups (11p15 LOM vs. controls) in completion times for TMT-A, a Bayesian Mann-Whitney *U*-test yielded a Bayes factor of BF_-0_ = 5.02 (moderate evidence), indicating that H1 is 5.02 times more likely to occur than the null hypothesis (H0). In other words, the 11p15 LOM group was 5.02 times more likely to be slower than the control group in completion times for TMT-A. The 11p15 LOM group was 2.97 times more likely to commit errors of commission compared to the control group (BF_-0_ = 2.97). However, this was considered “negligible” evidence for the alternative hypothesis (1 < BF < 3). Conversely, the Bayesian statistics showed moderate evidence in favor of the null hypothesis (no difference between the 11p15 group and the control group) for interference time of the Stroop test, total errors and perseverative errors of the MCST, and GEC, BRI, Inhibit, and Shift subscales of the BRIEF/BRIEF-A questionnaire (0.1 < BF < 0.33).

## Discussion

Previous studies have shown that individuals with SRS can have several difficulties related to attention and learning [39, 40, 43, 46, 49–52]. These difficulties may result from troubles in different cognitive domains and/or executive dysfunction. However, executive functioning has never been investigated, even though executive functions play an essential role in psychological and social development. Their dysfunction can have many repercussions in daily life. The present study was designed to establish the executive functioning of individuals with SRS due to 11p15 LOM or UPD(7)mat. Our aim was to determine which EFs were preserved and which ones were impaired in our SRS group, compared with the control group. Participants with SRS were expected to perform more poorly on executive tasks compared to a matched control group.

In contrast with this hypothesis, using Bayesian statistics, we showed that participants with SRS, specifically those with 11p15 LOM, showed similar performances to the control group. Only one difference has been found: the time to complete part A of the TMT appeared to be longer in the 11p15 LOM group compared to the control group. Therefore, the cognitive profile of individuals with SRS did not appear to be characterized by executive dysfunction. Nevertheless, we observed considerable inter-individual heterogeneity in SRS groups. Eight participants with SRS especially had clinically significant scores on at least three different EFs tasks. Two of the eight had been diagnosed with ADD/ADHD as children. These results may support the executive and attention difficulties observed in previous studies of individuals with clinical SRS. Indeed, as early as 1954, Russell reported cases of children showing signs of hyperactivity [75]. Subsequently, other researchers have reported attention/concentration and impulsivity difficulties [46], hyperactivity [39], and ADD/ADHD [49–51] have been observed in a few children with SRS. Thus, individuals with SRS could be at elevated risk of developing learning disabilities [76], or another neurodevelopmental disorder such as autism spectrum disorder [45] or ADD/ADHD.

On the other hand, the majority of individuals with clinical scores were adults with 11p15 LOM. This observation could be explained by random recruitment, but it could also be explained by other factors. Indeed, in some genetic syndromes such as 22q11.2 deletion syndrome [77], cognitive, behavioral and psychiatric phenotypes emerge across age. In the SRS, the cognitive profile could evolve with age and/or the presence of metabolic comorbidities. A high prevalence of metabolic disorders has also been reported in individuals with LOM 11p15 [78]. Individuals with SRS tend to develop Type 2 diabetes mellitus relatively early. In our group, two adults had Type 2 diabetes mellitus, while three others presented a risk of prediabetes, with insulin resistance or high fasting blood glucose level [40]. Type 2 diabetes mellitus may be associated with impaired cognitive function [79, 80]. It might be interesting to perform a longitudinal follow-up of individuals with SRS to better understand the executive functioning of participants and the potential impact of metabolic disorders on their cognitive profile.

In this study, some limitations need to be considered. The first limitation was the small number of participants with SRS (16 participants with 11p15 LOM and 3 with UPD(7)mat) and participants without SRS (*N =* 19). This problem is well-known in the field of rare diseases, the usual statistical tests and metrics being more appropriate for common diseases. Due to the rarity of the UPD(7)mat, our sample size here was much smaller and statistical comparisons between this group and the others were not appropriate. Therefore, statistical comparisons were only conducted between the 11p15 LOM and control groups. Future studies with larger sample sizes, particularly with more participants with UPD(7)mat, could confirm and generalize our results. Larger samples will also allow statistical comparisons between SRS groups (11p15 LOM vs. UPD(7)mat) needed to better understand phenotype-genotype relationships. The second limitation of this study is the existence of a potential representativeness bias. Indeed, our samples were composed of small groups of participants with SRS who volunteered to participate. The risk is that they are not representative of the general population with SRS. Indeed, we may have an over-representation of participants with or without difficulties in our study. For example, participants who volunteered for our study may have more difficulties than their peers and may seek support through study participation. Conversely, they may also have fewer difficulties and thus be more willing to participate in this study than other SRS patients. The third limitation was that this exploratory study had a cross-sectional design. A longitudinal study would provide a better understanding of the development of participants’ executive functions. This would include typical or atypical development, evolution, and especially unusual decline with age or associated diseases. Our methodology also had limitations, but can still guide future research. Thus, although our research design included a large number of executive tasks, not all modalities could be assessed and other tasks might also be more relevant. For example, while some authors consider the Digit span subtest to have an updating component, other tests may be more relevant to assess it, such as the *n*-Back task [81, 82]. Future studies could also investigate visuospatial working memory using the Corsi block-tapping test, attention in both auditory and visuospatial modalities with the Test of Everyday Attention, planning with the Tower of London, and motor inhibitory control with the Go/No-Go task [83–86]. Nevertheless, these highly structured paper-and-pencil tests are also open to criticism [87]. A more ecological and multifactorial assessment might be preferable, such as the Behavioural Assessment of the Dysexecutive Syndrome (BADS) [88]. Further studies remain mandatory to better understand the executive functioning of individuals with SRS and could lead to a better understanding of the syndrome and its cognitive profile. Such studies could also provide professionals with the tools to give more tailored support to individuals with SRS.

In summary, this first exploratory study investigates executive functioning in individuals with SRS, including adolescents and adults with 11p15 LOM or UPD(7)mat. The cognitive phenotype of SRS did not appear to be characterized by executive dysfunction. On the contrary, the overall the performance on executive tasks was similar between 11p15 LOM and control groups. Nevertheless, at the clinical level, several participants with SRS had clinically significant scores on several measures of EFs. Thus, individuals with SRS could be at high risk of developing executive dysfunction, or ADD/ADHD. Further studies remain necessary to confirm our findings. Future research should also investigate other cognitive domains which could explain difficulties reported in prior literature and by patients with SRS. Such studies have the potential to shed light on the profile of SRS and guide neuropsychological assessments in clinical practice.
